# Predicting Cardiovascular Events with Time-Lagged Inflammatory Dynamics: Stochastic Delay Modeling

**DOI:** 10.34133/csbj.0005

**Published:** 2026-03-18

**Authors:** Alexandre Vallée

**Affiliations:** Department of Epidemiology and Public Health, Foch Hospital, Suresnes 92150, France.

## Abstract

**Background:** Inflammatory responses are often delayed, nonlinear, and subject to stochastic variability, features typically absent from conventional cardiovascular events models. This study investigates whether delayed inflammatory dynamics, simulated via stochastic delay differential equations (SDDEs), can predict cardiovascular events and enrich digital twin architectures. **Methods:** A mathematical model was developed to simulate interactions between 3 biomarkers, neutrophils (rapid), C-reactive protein (CRP; intermediate), and albumin (slow negative regulator), under delayed and stochastic dynamics. Using an SDDE framework, 100 virtual individuals were simulated. Cardiovascular event was defined as exceeding a threshold in a latent clinical output *Y*(*t*), representing a composite cardio-inflammatory stress state. In parallel, proxy delay ratios (CRP/neutrophils and CRP/albumin) were derived in the UK Biobank cohort (*n* = 502,478) as structural analogs of the simulated delays to assess epidemiological consistency with the model’s predictions over 11.9 years of follow-up. **Results:** Simulated individuals with delayed symptom peaks (Time_to_Peak_Y) showed a strong association with cardiovascular events (20% prevalence): Delayed responders were disproportionately associated with simulated events. Stochastic noise introduced interindividual variability, and temporal delay emerged as a strong model-internal discriminator of trajectories within the SDDE framework. In UK Biobank, delay-based ratios were significantly associated with incident heart failure (*P* < 0.001), even after multivariable adjustment. Kaplan–Meier curves showed early risk separation across delay tertiles. **Conclusion:** Delay-aware inflammatory dynamics offer a powerful lens to simulate disease trajectories in digital twins. SDDE-based models capture both timing and regulatory imbalance, bridging mechanistic simulation and real-world epidemiological consistency. This framework strengthens the potential of digital twins to deliver personalized risk modeling.

## Graphical Abstract

**Figure:**
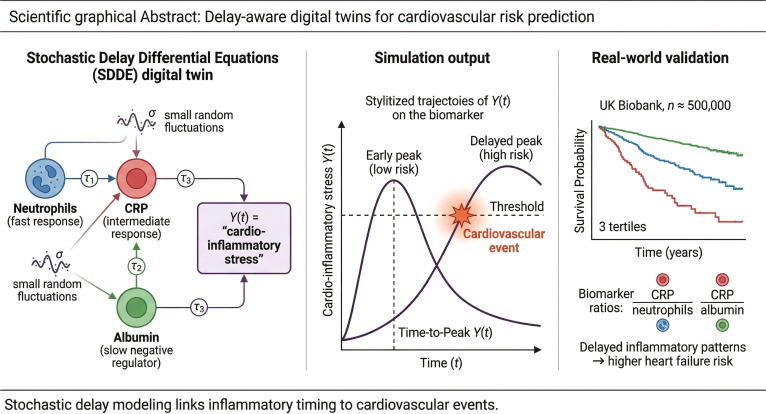

## Introduction

Digital twins in medicine are computational models designed to simulate the dynamic and individualized evolution of health states, integrating physiological data, treatment history, and environmental exposures [1]. These models hold great promise for enabling real-time prediction of disease trajectories, guiding therapeutic decisions, and supporting personalized care across clinical contexts [2–4]. It is important to distinguish between operational clinical digital twins and conceptual digital twin frameworks [5]. Operational digital twins are designed for real-time clinical deployment, requiring individual-level parameterization, continuous data assimilation, prospective validation, and direct integration into clinical decision-making [6]. In contrast, the present work adopts a conceptual digital twin approach, in which mathematical models are used as theoretical instruments to explore system-level behavior, generate mechanistic hypotheses, and interrogate how specific dynamical features, such as delay and stochasticity, may structure risk trajectories. The stochastic delay differential equation (SDDE)-based digital twin proposed here is therefore not intended as a deployable clinical tool but as a framework for hypothesis generation and model-driven interpretation of complex physiological dynamics.

However, most digital twin frameworks rely on deterministic and instantaneous models that often fail to reflect the inherent complexity of biological systems. In reality, physiological responses to interventions are rarely immediate and frequently display both temporal delays and stochastic variability. For example, the secretion of stress-related hormones, the immune system’s response to infection, or the effect of a pharmacological agent often occurs after a latent period and varies markedly across individuals, even under similar conditions [7–9].

These phenomena highlight 2 critical features often overlooked in standard modeling approaches: delay and stochasticity [10,11]. Delays arise from biological processing times, such as signal transduction, gene expression, and feedback inhibition, while stochasticity reflects the influence of unmeasured or random perturbations, such as genetic background [12], circadian rhythms [13], or microenvironmental factors [14].

SDDEs provide a natural and powerful framework to model such systems [15]. Unlike ordinary differential equations, SDDEs allow both memory (delays) and randomness (noise) to be explicitly incorporated, making them particularly suitable for representing multiscale biological phenomena [16]. In this study, we propose an SDDE-based digital twin architecture to capture delayed inflammatory dynamics and investigate their capacity to predict adverse cardiovascular outcomes.

Using both synthetic simulations and real-world data from the UK Biobank, we demonstrate that delayed interactions between biomarkers (e.g., neutrophils, C-reactive protein [CRP], and albumin) are not merely descriptive features but mechanistically informative for exploring individual risk trajectories. These findings suggest that time-lagged inflammatory responses, especially when coupled with regulatory imbalance, may serve as a novel and clinically meaningful dimension of cardiovascular risk modeling.

Thus, the objectives of this study are 3-fold: (a) to develop an SDDE model that simulates the dynamic interplay between inflammatory biomarkers, incorporating biologically plausible delays and stochastic fluctuations; (b) to investigate the system-level role of delayed inflammatory dynamics in generating heterogeneous latent clinical trajectories and structuring simulated cardiovascular event risk in a simulated population of virtual subjects; and (c) to validate the predictive value of inferred temporal delays using real-world longitudinal data from the UK Biobank cohort by assessing whether interbiomarker delay proxies are associated with incident heart failure, independent of conventional cardiovascular risk factors.

## Materials and Methods

### Model formulation of SDDEs

A digital twin can be defined as a computational model for an individual $\tau_{i}$, parameterized by their physiological state, treatment history, and environmental exposures. In this framework, the digital twin simulates the temporal evolution of internal biological markers and external clinical states.

In the following, the conceptual digital twin associated with individual *i* is denoted by $T_{i}$, whereas $\tau_{i}$ is reserved exclusively for temporal delay parameters in the SDDE system.

Within the digital twin framework adopted here, each component of the SDDE system corresponds to a distinct element of the twin architecture. The biomarker variables (neutrophils, CRP, and albumin) represent internal physiological state variables of the twin, encoding fast, intermediate, and slow inflammatory processes. The delay parameters (*τ* terms) capture latent physiological processing times and regulatory latencies intrinsic to immune signaling and feedback control. The stochastic terms model unobserved biological variability and environmental perturbations acting on the system. Finally, the output variable *Y*(*t*) corresponds to a latent clinical state of the digital twin, integrating inflammatory activation and regulatory imbalance into a composite cardio-inflammatory stress signal, rather than a directly observed symptom or biomarker.

Mathematically, a digital twin $T_{i}$ is expressed as a set of stochastic dynamical equations:

$$T_{i}:X_{i}\left(t\right)=\Phi_{i}\left(X_{i}\left(t^{\prime }\right),\;S_{i}\left(t^{\prime }\right),\;\xi_{i}\left(t^{\prime }\right)\right),\text{for}\;t^{\prime }\leq t,$$ (1)

where $X_{i}\left(t\right)$ is the state vector (e.g., biomarkers and symptoms) at time *t*, $S_{i}\left(t\right)$ is the treatment or perturbation function, $\xi_{i}\left(t\right)\;$encodes stochastic processes (e.g., Brownian motion), and $\Phi_{i}$ is an operator encoding personalized physiological dynamics with memory (i.e., delays).

To implement this, we propose a concrete instantiation using SDDEs. Let $S\left(t\right)$ denote the input stimulus and $B\left(t\right)$ denote the internal biomarker dynamics. *Y*(*t*) is a latent clinical state representing an integrated cardio-inflammatory stress burden. It does not correspond to a directly observed clinical symptom or a measured inflammatory biomarker but rather to a composite internal variable reflecting the downstream impact of inflammatory activation and regulatory imbalance.

$Y\left(t\right)$ is not a directly observed clinical symptom but a composite internal variable reflecting the downstream impact of inflammatory activation and regulatory imbalance.

The evolution of the system is modeled by the following SDDEs:

$$\mathrm{dB}\left(t\right)=\left[\alpha_{1}S\left(t-\tau_{1}\right)-\alpha_{2}S\left(t-\tau_{2}\right)\right]\;\mathrm{dt}+\sigma_{1}B\left(t-\tau_{2}\right)dW_{1}\left(t\right),$$ (2)

$$\mathrm{dY}\left(t\right)=\left[\beta_{1}B\left(t-\tau_{3}\right)-\beta_{2}Y\left(t-\tau_{3}\right)\right]\;\mathrm{dt}+\sigma_{2}Y\left(t-\tau_{3}\right)dW_{2}\left(t\right)$$ (3)

where $\alpha_{1}$ and $\alpha_{2}$ represent amplification and clearance rates for biomarkers; *β*_1_ and *β*_2_ describe symptom sensitivity and decay; $\tau_{1},\tau_{2},\text{and}\;\tau_{3}$ are the respective delays; and $\sigma_{1}\;\text{and}\;\sigma_{2}\;$ represent the noise intensities associated with each process. For simplicity and interpretability, the noise intensities $\sigma_{i}$ are assumed to be constant over time in the present implementation, reflecting stationary stochastic variability. Allowing time-dependent or state-dependent noise amplitudes is possible within the SDDE framework but was not pursued here to preserve model parsimony and focus on the role of delayed dynamics.

For consistency with the SDDE formulation, the clinical output is modeled as a delayed process; the dependence on *Y*(*t* − *τ*_3_) reflects the same regulatory latency introduced in the core SDDE system.

### Choice and interpretation of delay parameters (*τ*_1_ to *τ*_8_)

The delay parameters introduced in the SDDE system (*τ*_1_ to *τ*_8_) are not estimated from individual-level biological data nor intended to represent exact physiological time constants. Instead, they are conceptual parameters chosen to reflect biologically plausible relative time scales within an inflammatory cascade.

Specifically, delays were selected to preserve the known temporal ordering of immune processes:

1. Rapid innate responses (e.g., neutrophil activation following stress)
2. Intermediate downstream responses (e.g., CRP induction following cytokine signaling)
3. Slower regulatory or compensatory mechanisms (e.g., albumin suppression and recovery). This ordering is well documented in immunology and systems biology literature, even though precise interindividual delays vary widely [8,17].

The absolute values of *τ*_1_ to *τ*_8_ were therefore chosen for simulation purposes, ensuring separation of fast, intermediate, and slow processes, rather than to reproduce exact clinical kinetics. The model is intended as a conceptual digital twin framework, designed to explore how delayed interactions and stochasticity structure system-level dynamics, rather than as a calibrated physiological model [18].

Consequently, simulation time is expressed in abstract units, and delays should be interpreted relatively rather than as direct mappings to clock time. Future extensions of the framework could incorporate data-driven or individualized delay estimation when longitudinal biomarker measurements are available. The existence and ordering of such delays in inflammatory and physiological systems are well established in the literature, even though their exact magnitudes vary across individuals and contexts [8,17,18]. SDDEs provide a recognized framework to explore the system-level consequences of such delays without requiring precise parameter calibration [18].

### Choice and role of model parameters (*α_i_*, *β_i_*, *σ_i_*)

The parameters *α_i_*, *β_i_*, and *σ_i_* introduced in the SDDE system were not estimated from empirical biological data nor directly taken from previous quantitative studies. Instead, they were manually specified within a conceptual and exploratory modeling framework, following common practice in stochastic and delayed modeling of biological systems [15,19].

The *α_i_* parameters represent amplification or production terms governing the strength of biomarker activation, whereas the *β_i_* parameters correspond to clearance, decay, or negative feedback processes ensuring bounded and stable dynamics [18]. The *σ_i_* parameters control the magnitude of stochastic fluctuations and represent unobserved biological variability and environmental perturbations acting on the system [20,21].

Parameter values were selected to satisfy 3 criteria:

1. Stability of the system in the absence of external perturbation
2. Emergence of nontrivial transient dynamics under delayed feedback
3. Sufficient stochastic variability to generate heterogeneous trajectories across virtual individuals exposed to identical inputs.

No attempt was made to calibrate or identify parameters against real-world data, as the primary objective of the model is mechanistic exploration rather than quantitative prediction, consistent with the conceptual digital twin framework adopted in this study [5,18].

Accordingly, parameters should be interpreted as structural and dimensionless quantities defining the qualitative behavior of the system rather than as physiologically measurable rates. Future work combining SDDE models with longitudinal biomarker measurements could allow data-driven or individualized parameter estimation. The use of linear terms and manually specified parameters was intentional to preserve model parsimony and to isolate the specific contribution of delayed feedback and stochasticity, without introducing additional nonlinear mechanisms. Within this framework, linear dynamics should be interpreted as a local approximation of more complex immune processes rather than as a full physiological representation.

### Delayed stochastic noise

In contrast to classical stochastic differential equations where noise is applied instantaneously, delayed stochastic noise refers to stochastic perturbations that depend on a past system state. Formally, the noise term takes the form:

$$\sigma \left(t-\tau \right)X\left(t-\tau \right)\mathrm{dW}\left(t\right)$$ (4)

where *τ* > 0 is the delay, $X\left(t-\tau \right)$ is the system state at the earlier time (*t* −τ), and *dW*(*t*) is a standard Wiener increment.

### Interpretation and biological meaning

The rationale for the proposed structure is rooted in the biological observation that responses to perturbations are rarely immediate and often display intrinsic variability that depends on prior states. The delay $\tau_{1}\;$between the administration of a stimulus $S\left(t\right)\;$and the subsequent activation of a biomarker $B\left(t\right)$ mirrors phenomena such as the delayed secretion of cortisol following acute stress or the lagged onset of pharmacological effects posttreatment. Internally, biomarkers themselves are governed by regulatory feedback loops, often operating with intrinsic delays $\tau_{2}$, such as negative feedback in immune cytokine signaling pathways. Similarly, the onset of clinical symptoms $Y\left(t\right)\;$following biomarker fluctuations often occurs after a physiological processing period, captured by the delay $\tau_{3}$.

Furthermore, biological systems are inherently stochastic, and the variability in responses is not merely noise applied to instantaneous states but often a consequence of historical conditions. Delayed stochastic noise terms, such as $\sigma_{1}B\left(t-\tau_{2}\right)dW_{1}\left(t\right)\;$ and $\sigma_{2}Y\left(t-\tau_{3}\right)dW_{2}\left(t\right)$, model how random fluctuations manifest with dependence on prior biomarker or clinical states. This approach reflects observed phenomena like delayed bursts of cytokines or variability in delayed pharmacodynamic responses.

By integrating these features, the SDDE framework realistically models the dynamics of therapeutic latency, symptom onset, and intraindividual variability across time scales. The model thus supports digital twins that are capable of capturing not only the mean expected trajectories but also the stochastic and delayed nature of biological responses critical for individualized simulation and prediction.

In real biological systems, neutrophil responses typically occur within hours, CRP rises over several hours to days, and albumin changes reflect slower processes occurring over days; however, the present model encodes these dynamics in relative rather than absolute time units.

### Numerical methods and simulation

To explore the mechanistic impact of delayed and stochastic inflammatory responses, we developed an SDDE model involving 3 key biomarkers: neutrophils, CRP, and albumin. These biomarkers were selected on the basis of their established roles in the temporal cascade of systemic inflammation:

• Neutrophils represent a rapid, first-line immune response to stress or insult.
• CRP is a downstream acute-phase reactant that rises in response to proinflammatory cytokines such as interleukin-6, with a moderate delay.
• Albumin is a negative acute-phase protein, typically decreasing in inflammatory states, and reflects homeostatic compensation.

**Fig. 1.**
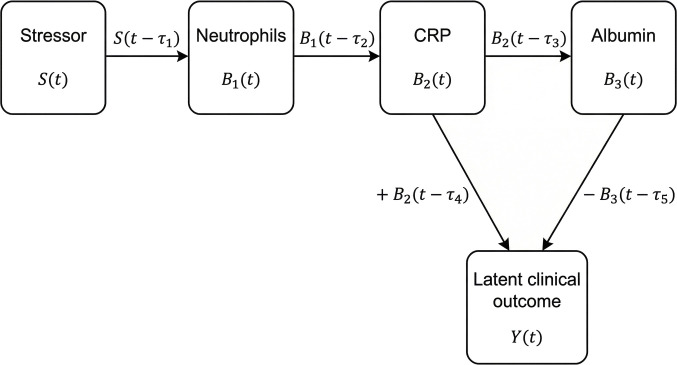
Schematic representation of delayed inflammatory regulation and clinical outcome modeling. Diagram illustrating the delayed cascade linking neutrophils, CRP, and albumin to a downstream clinical outcome *Y*(*t*). Neutrophils activate CRP with a temporal delay, and both CRP (positively) and albumin (negatively) contribute to the symptom trajectory. Albumin is regulated by neutrophils with a separate delay. Each interaction is subject to time lag *τ*, representing biological delays in activation or suppression. This structure serves as the basis for the SDDE model used to simulate cardiovascular risk dynamics.

The model simulates a stressor signal $S\left(t\right)\;$that triggers neutrophilic response $B_{1}\left(t\right)$, which, in turn, stimulates CRP $B_{2}\left(t\right)$ and subsequently suppresses albumin $B_{3}\left(t\right)$. The clinical outcome $Y\left(t\right)\;$is driven by both CRP (positively) and albumin (negatively), representing the interplay of proinflammatory and regulatory forces (Fig. 1).

The SDDE system is defined as follows:

$$\begin{array}{c} dB_{1}\left(t\right)=\\ \left[\alpha_{1}S\left(t-\tau_{1}\right)-\alpha_{2}B_{1}\left(t-\tau_{2}\right)\right]\;\mathrm{dt}+\sigma_{1}B_{1}\left(t-\tau_{2}\right)dW_{1}\left(t\right), \end{array}$$ (5)

$$\begin{array}{c} dB_{2}\left(t\right)=\\ \left[\gamma_{1}B_{1}\left(t-\tau_{3}\right)-\gamma_{2}B_{2}\left(t-\tau_{4}\right)\right]\;\mathrm{dt}+\sigma_{2}B_{2}\left(t-\tau_{4}\right)dW_{2}\left(t\right), \end{array}$$ (6)

$$\begin{array}{c} dB_{3}\left(t\right)=\\ \left[\theta_{1}-\theta_{2}B_{2}\left(t-\tau_{5}\right)-\theta_{3}B_{3}\left(t-\tau_{6}\right)\right]\;\mathrm{dt}+\sigma_{3}B_{3}\left(t-\tau_{6}\right)dW_{3}\left(t\right), \end{array}$$ (7)

$$\begin{array}{c} \mathrm{dY}\left(t\right)=\\ \left[\beta_{1}B_{2}\left(t-\tau_{7}\right)-\beta_{2}B_{3}\left(t-\tau_{8}\right)-\beta_{3}Y\left(t-\tau_{9}\right)\right]\;\mathrm{dt}+\sigma_{4}Y\left(t-\tau_{9}\right)dW_{4}\left(t\right) \end{array}$$ (8)

Each equation includes a delay term $\tau_{i}$ representing biologically plausible latencies: stress-to-neutrophil activation ($\tau_{1}$), neutrophil-to-CRP induction ($\tau_{3}$), CRP-to-albumin suppression ($\tau_{5}$), and propagation delays to the clinical outcome ($\tau_{7}$, $\tau_{8}$*,* $\tau_{9}$).

The parameter *θ*_1_ represents a baseline production or homeostatic input for the *B*_3_ compartment, capturing persistent physiological or metabolic contributions that are not explicitly driven by upstream inflammatory signals. This term ensures a nonzero equilibrium level of *B*_3_ in the absence of external stimulation.

Simulations were initialized with low baseline values for all variables. Noise terms $\sigma_{i}dW_{i}\left(t\right)$ represent biological variability. For each synthetic subject, we extracted trajectory metrics including the following:

• Time-to-peak *Y*(*t*)
• Maximum *Y*(*t*)
• Occurrence of a cardiovascular event: Event_CV = 1 if max*Y*(*t*) > 1.5

Accordingly, Event_CV was set to 1 when max*Y*(*t*) exceeded the predefined threshold of 1.5 and to 0 otherwise [i.e., when max*Y*(*t*) ≤ 1.5].

Each biomarker was modeled as a node in a delayed regulatory cascade, with neutrophils driving CRP production and CRP subsequently suppressing albumin. The downstream clinical outcome, denoted as *Y*(*t*), represents a latent cardio-inflammatory stress signal integrating proinflammatory (CRP) and regulatory (albumin) influences. It is not a symptom score nor a direct measure of inflammatory burden. The system includes biologically plausible delay terms for each interaction, as well as multiplicative and additive stochastic noise to capture interindividual variability.

The model was implemented using an Euler–Maruyama integration scheme adapted for SDDEs, and simulations were run over a time window of 100 units. A total of 100 virtual individuals were generated and exposed to the same input stressor but were subject to random variation in dynamic trajectories. To generate a dataset with a realistic distribution of clinical outcomes, a cardiovascular event was defined as occurring when the peak of *Y*(*t*) exceeded a predefined threshold. The threshold was empirically calibrated such that exactly 20% of subjects (*n* = 20) were labeled as Event_CV = 1. This balancing approach allows for meaningful statistical comparison between event and nonevent groups while preserving the stochastic structure of the model. The threshold was deliberately calibrated to achieve a predefined event prevalence (20%) in the simulated population rather than arising from stochastic variation. Although the event threshold is not derived from a specific clinical trial, it was empirically validated within the simulated population to ensure a realistic distribution of cardiovascular events and to reflect heterogeneity in latent disease trajectories rather than being arbitrarily user-defined.

The SDDE system was numerically integrated using an Euler–Maruyama scheme adapted for SDDEs. Simulations were performed using a fixed time step Δ*t*, chosen sufficiently small to ensure numerical stability and smooth trajectories over the simulation horizon.

Delayed state values [e.g., *X*(*t* − *τ*)] were obtained by storing past states in memory and retrieving the corresponding values via discrete indexing based on the delay length and time step. For time points *t* < max(*τ*), initial history functions were defined by constant baseline values for all state variables.

Numerical stability was assessed empirically by verifying that all simulated trajectories remained bounded and free of numerical artifacts (e.g., divergence or oscillations driven by discretization) across the entire simulation window. Additional sensitivity analyses using alternative thresholds and noise levels confirmed that the qualitative behavior of the system was robust to moderate numerical perturbations.

Because the cardiovascular event is defined endogenously as a threshold exceedance of the simulated output *Y*(*t*), the resulting association between Time_to_Peak_Y and Event_CV is structurally induced by the model. The observed separation therefore reflects an emergent property of the SDDE system rather than an empirical validation of biological determinism. Accordingly, the simulation results are not intended to represent predictive performance but to illustrate theoretical properties of delayed stochastic systems under idealized conditions.

**Fig. 2.**
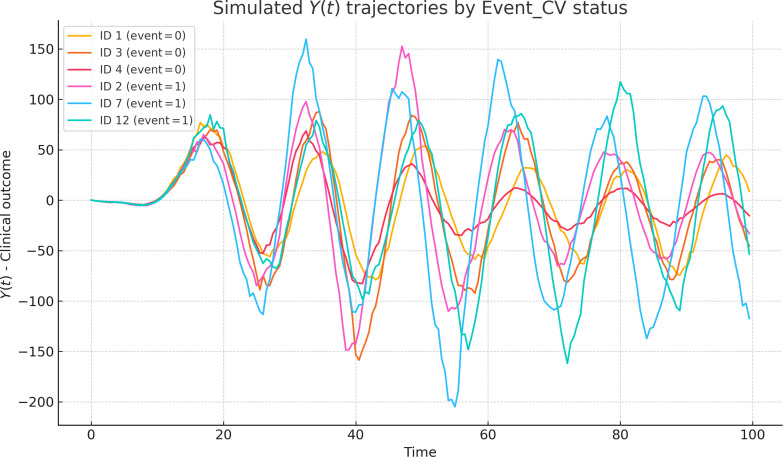
Simulated *Y*(*t*) trajectories by cardiovascular event status. Simulated trajectories of the latent clinical output *Y*(*t*) for 6 representative virtual individuals. Curves are displayed on the raw *Y*(*t*) scale to illustrate qualitative differences in trajectory shape and timing. Cardiovascular event status (Event_CV) is defined on the basis of the maximum of a normalized version of *Y*(*t*); therefore, the event threshold is not shown in this figure and should not be interpreted directly from the absolute trajectory values. These simulation results are intended to illustrate theoretical properties of the SDDE framework under idealized conditions and should not be interpreted as direct clinical or epidemiological validation.

To illustrate the behavior of the SDDE system, the trajectories of *Y*(*t*) for 6 representative subjects have been plotted in Fig. 2. Despite identical inputs and model parameters, the interplay of delays and noise produced markedly different clinical trajectories. Subjects without simulated cardiovascular events (Event_CV = 0) displayed earlier and more moderate peaks, while those with Event_CV = 1 exhibited delayed and often more amplified responses. These variations highlight the capacity of delayed stochastic systems to generate clinically relevant heterogeneity in simulated trajectories in disease expression, even in the absence of parameter tuning.

Such heterogeneity mirrors real-world clinical trajectories, where symptom onset following an inflammatory insult can vary in both timing and severity. These dynamics justify the use of time-to-peak *Y*(*t*) as a mechanistically grounded feature for downstream risk prediction. Figure 2 reinforces the SDDE model’s capacity to generate both physiologically plausible and clinically informative output behaviors.

Table S1 summarizes the individual-level simulation outputs [max*Y*(*t*), Time_to_Peak_Y, and Event_CV], supporting the hypothesis that biological timing, not just biomarker levels, governs vulnerability in dynamic pathophysiological systems.

This model architecture allows for multiscale and antagonistic dynamics, capturing both early activation and compensatory regulatory feedback. It thus reflects a physiologically plausible scenario to explore how response delays and imbalance among inflammatory mediators may drive clinical outcomes.

To assess numerical robustness, selected simulations were repeated with a smaller time step (e.g., Δ*t*/2), yielding qualitatively similar trajectories and delay-based separation, suggesting limited sensitivity to discretization.

### Software implementation

All SDDE simulations were implemented using Python, whereas statistical analyses of the UK Biobank cohort were performed using SAS software (version 9.4; SAS Institute, Cary, NC, USA). The simulation code was developed for conceptual and exploratory modeling purposes and is not publicly released at this stage.

### Sensitive analyses

To assess the robustness of the observed association between temporal delay and simulated cardiovascular events, we conducted a series of sensitivity analyses addressing event definition, outcome noise, and interindividual parameter variability.

First, the event threshold defining “Event_CV” was varied to generate alternative event prevalences (15%, 20%, and 30%), corresponding to different quantiles of the “max*Y*(*t*)” distribution. For each threshold, the association between “Time_to_Peak_Y” and event status was reassessed.

Second, independent outcome noise was introduced at the event definition stage. Specifically, an observed outcome was defined as

$${\mathrm{Max}}_{Y}^{\mathrm{obs}}={\mathrm{Max}}_{Y}+\varepsilon =$$ (9)

where $\varepsilon ∼N\left(0,\;\sigma^{2}\right)$ with σ set to 10 to 20% of the standard deviation (SD) of max*Y*(*t*). Event status was then recalculated on the basis of ${\mathrm{Max}}_{Y}^{\mathrm{obs}}$.

Third, moderate interindividual heterogeneity was introduced by allowing selected model parameters [delay and gain terms linking CRP to the clinical outcome *Y*(*t*)] to vary across individuals, drawn from normal distributions centered on the nominal values with 10% coefficient of variation.

Additional robustness checks showed that increasing stochastic variability attenuated near-complete stratification but preserved the qualitative relationship between delayed trajectories and event emergence (Figs. S1 and S2).

### Simulation analysis: Delayed dynamics in a multimarker regulatory cascade

#### Simulation results: SDDE-based mechanistic exploration

We extended the SDDE model to simulate a multiscale inflammatory cascade involving 3 interacting biomarkers: neutrophils (rapid innate response), CRP (intermediate-phase reactant), and albumin (negative acute-phase protein reflecting compensatory regulation). Each was modeled with biologically plausible delay terms and stochastic noise. The outcome variable *Y*(*t*) was defined as a function of proinflammatory (CRP) and regulatory (albumin) signals.

Using this framework, we simulated 100 virtual subjects over a 100-unit time horizon. For each subject, we computed the following:

• The full trajectory of *Y*(*t*)
• The maximum value max *Y*(*t*) [max *Y*(*t*)]
• The time to peak $Y\left(t\right)$ (i.e., Time_to_Peak_Y)
• A binary event variable Event_CV was defined: 1 if max*Y*$\left(t\right)$ > 1.5, 0 otherwise.

Of 100 simulated individuals, 20 experienced a cardiovascular event based on the SDDE-derived threshold [Event_CV = 1 if max*Y*(*t*) > 1.5]. Event-positive subjects exhibited systematically longer delays in reaching the peak of their symptom trajectory *Y*(*t*), with a mean Time_to_Peak_Y of 34.2 compared to 26.8 in event-free individuals. Group differences in Time_to_Peak_Y were additionally assessed using nonparametric tests, confirming a statistically significant separation between event and nonevent trajectories (*P* < 0.001). Given the deterministic structure of the simulated outcome, formal hypothesis testing was considered complementary rather than central to the analysis. The distribution of max*Y*(*t*) was also right-shifted in the event group, suggesting that both the magnitude and the timing of symptom amplification are mechanistically linked to risk emergence. By construction, the distribution of max*Y*(*t*) is necessarily right-shifted in the event group, as the event definition itself is based on threshold exceedance. Accordingly, max*Y*(*t*) is not interpreted as a predictive or explanatory variable of risk. Instead, the analysis focuses on how risk emerges from the temporal dynamics leading to threshold crossing, particularly the timing, shape, and delayed structure of *Y*(*t*) trajectories preceding the event.

**Fig. 3.**
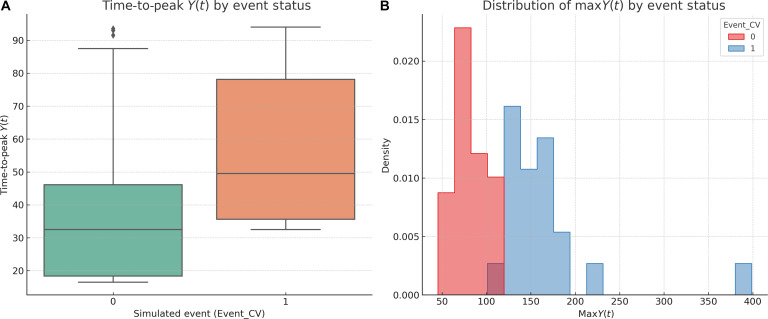
Delayed latent symptom response and event risk in the 3-biomarker SDDE simulation. Composite figure illustrating the temporal and amplitude characteristics of the clinical outcome *Y*(*t*) across simulated individuals. (A) Box plot of time-to-peak *Y*(*t*) (Time_to_Peak_Y), stratified by event status (Event_CV = 0 versus 1). Individuals who experienced a simulated cardiovascular event displayed markedly longer delays in symptom escalation. (B) Histogram of max*Y*(*t*) values, showing a rightward shift in peak intensity among event-positive subjects. Together, these findings confirm that delayed and amplified symptom responses, emerging from feedback-regulated, stochastic biomarker interactions, are strongly associated with adverse outcomes in the SDDE simulation framework. These simulation results are intended to illustrate theoretical properties of the SDDE framework under idealized conditions and should not be interpreted as direct clinical or epidemiological validation.

Figure 3A (left) demonstrates a clear stratification of Time_to_Peak_Y across event status using a box plot, while Fig. 3B (right) shows a visibly distinct distribution of max*Y*(*t*) between the 2 groups. These patterns reflect the delayed and deregulated interplay between inflammatory signals (neutrophils and CRP) and compensatory regulation (albumin) encoded in the SDDE system.

A standard logistic regression using Time_to_Peak_Y as the sole predictor failed to converge due to near-complete stratification, a phenomenon that reflects the structural properties of the SDDE system under a calibrated event definition rather than overfitting or real-world predictive determinism: Individuals with more delayed peaks almost exclusively developed simulated events, while early responders remained event-free. This reinforces the concept that temporal delay, even under stochastic noise and feedback, can serve as a strong model-internal discriminator under the SDDE construction in biologically inspired systems.

**Fig. 4.**
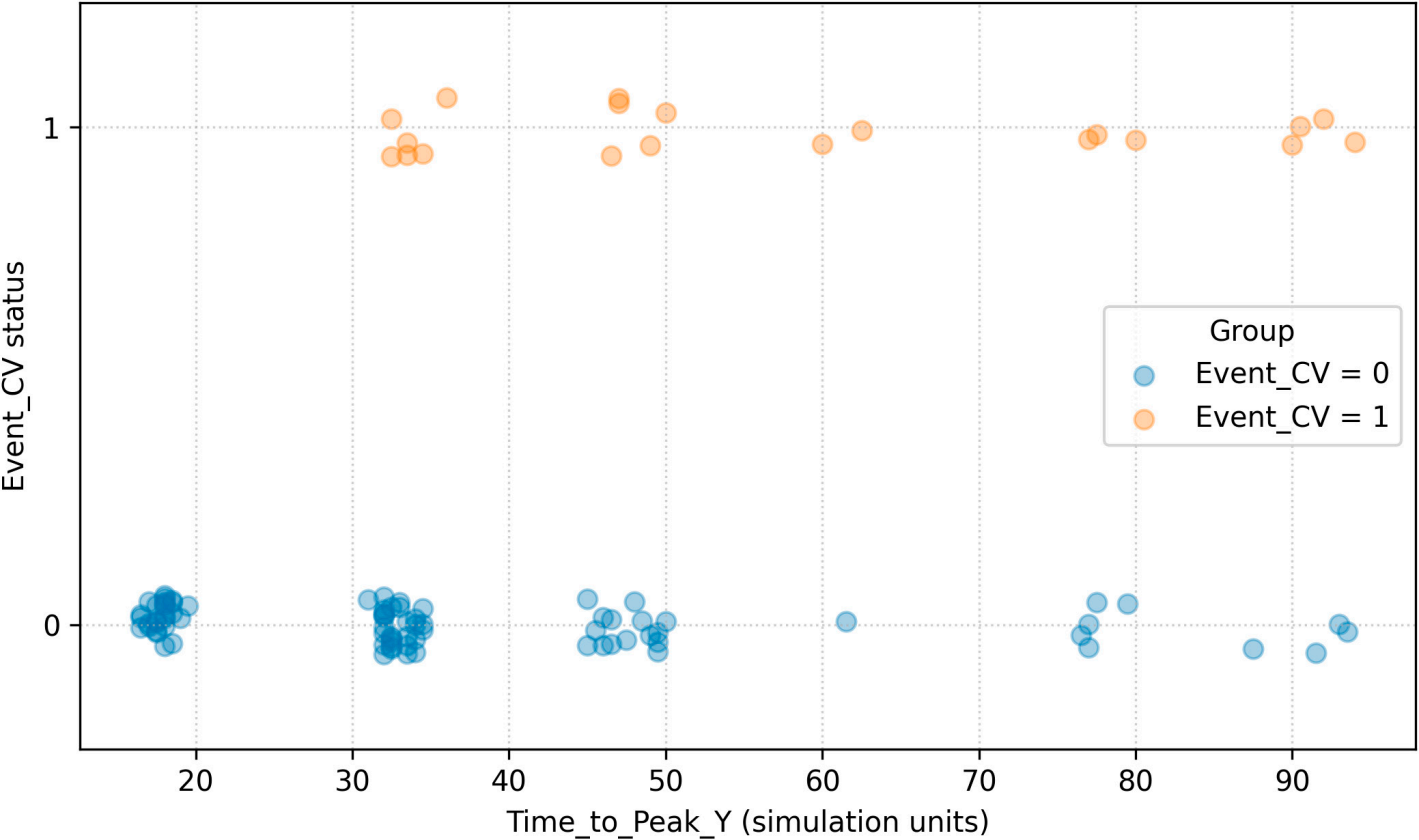
Relationship between time-to-peak *Y*(*t*) and simulated cardiovascular event status in the SDDE framework. Scatter plot showing the relationship between Time_to_Peak_Y and simulated Event_CV across 100 virtual individuals. Each point represents one subject. To mitigate overplotting, points are displayed with partial transparency and a small vertical jitter around Event_CV = 0 and Event_CV = 1; darker regions therefore indicate higher point density resulting from overlapping observations. The observed stratification reflects a theoretical property of the SDDE system under fixed parameters and an empirically calibrated event definition. It should not be interpreted as predictive performance or biological determinism but rather as an illustration of how delayed dynamics can structure the emergence of event versus nonevent trajectories within the model. These simulation results are intended to illustrate theoretical properties of the SDDE framework under idealized conditions and should not be interpreted as direct clinical or epidemiological validation.

Figure 4 demonstrates that Time_to_Peak_Y functions as an almost perfectly discriminative variable for cardiovascular event risk in the SDDE simulation. A distinct separation is observed: Individuals with earlier symptom peaks uniformly avoided events (Event_CV = 0), whereas those with delayed responses consistently developed them (Event_CV = 1). The near-complete stratification observed here is a theoretical property of the SDDE construction under a calibrated event definition and fixed parameters and should not be interpreted as predictive performance or biological determinism.

These findings strengthen the central hypothesis that temporal misalignment in inflammatory and regulatory cascades, as represented by biomarker delays (neutrophils → CRP → albumin), has mechanistic significance. The SDDE framework offers a physiologically grounded and mathematically explicit approach for modeling such latent delays, reinforcing the potential of time-aware features to improve risk prediction in future digital twin architectures.

### Sensitivity analyses of delay-based separation

The association between Time_to_Peak_Y and simulated cardiovascular events remained robust across alternative event definitions. When the event threshold was varied to generate event prevalences of 15%, 20%, and 30%, delayed responders consistently exhibited a higher probability of events. However, the sharpness of separation decreased, as the threshold moved away from the calibrated 20% prevalence, indicating that near-complete stratification is contingent on the specific event definition (Table S2).

When independent outcome noise was introduced at the event definition stage, near-complete stratification was no longer observed. Nevertheless, Time_to_Peak_Y remained a strong discriminator of event risk, with delayed trajectories showing substantially higher event probabilities compared to early responders (Fig. S1).

Finally, introducing moderate interindividual variability in delay and gain parameters reduced the determinism of the model while preserving its qualitative behavior. Under parameter heterogeneity, delayed trajectories remained disproportionately associated with simulated events, although with increased overlap between event and nonevent groups (Fig. S2).

Together, these sensitivity analyses demonstrate that near-complete stratification is an emergent property of the SDDE construction under fixed parameters and calibrated outcomes. Importantly, they also show that the core finding, the relevance of temporal delay as a risk-defining feature in delayed stochastic systems, persists under noise, threshold variation, and parameter heterogeneity. Under perturbed conditions, receiver operating characteristic analysis yielded moderate discriminative performance rather than near-deterministic separation (area under the curve ≈ 0.75 to 0.82), and misclassification rates increased substantially, with both false-positive and false-negative classifications observed.

In real clinical settings, near-complete stratification is neither expected nor desirable, as it would imply an implausible absence of biological heterogeneity, measurement error, and unobserved confounding.

Importantly, the following epidemiological analyses are presented independently from the simulation results and are intended to assess real-world consistency with the SDDE-derived hypotheses rather than to validate the simulated dynamics directly.

## Results

### Data analysis: Real-world validation of delayed response

#### UK Biobank population

The UK Biobank stands as a forward-looking cohort initiative aimed at investigating, preventing, diagnosing, and treating chronic diseases, notably cardiovascular disease (CVD) in adults [22–24]. This extensive study encompassed 502,478 individuals from across 22 cities in the UK, all registered with the UK National Health Service. Participants were aged between 40 and 69 years when they joined UK Biobank between 2006 and 2010. In this cohort, participants were thoroughly phenotyped and genotyped. This process involved their responses to detailed questionnaires, participation in computer-assisted interviews and various physical and functional assessments. In addition, the collection of biological samples including blood, urine, and saliva was integral to the study [25].

The data gathered in the UK Biobank encompass a wide range of factors. These include socioeconomic variables, behavioral and lifestyle information, a comprehensive mental health assessment, and clinical diagnoses and treatments. Furthermore, the cohort has been a rich source of genetic information, alongside imaging and physiological biomarkers obtained from blood and urine samples. The protocol that guided this extensive collection and analysis of data is thoroughly documented in the scientific literature [26].

#### Ethical considerations

All participants in the study provided their informed consent electronically. The UK Biobank obtained ethical approval from the North West Multi-centre Research Ethics Committee, which extends its jurisdiction across the entire UK. The study adhered to the principles outlined in the Declaration of Helsinki and received approval from the Northwest-Haydock Research Ethics Committee. The specific protocol code for this approval was 21/NW/0157, and the date of approval was 2021 June 21. For more detailed information, see https://www.ukbiobank.ac.uk/learn-more-about-uk-biobank/about-us/ethics.

#### Methods

We used baseline data from over 502,478 participants in the UK Biobank to test whether an inferred delay between psychological stress and inflammatory response could predict incident cardiovascular outcomes.

We sought to replicate the logic of the SDDE model by testing whether the imbalance and temporal misalignment between these biomarkers, particularly between upstream (neutrophils) and downstream (CRP and albumin) signals, could predict cardiovascular outcomes (i.e., heart failure).

To do this, we constructed composite-delay-like proxy variables, intended as structural analogs of delayed inflammatory dynamics rather than direct measurements of physiological time lags, including the following:

$$\tau_{\text{composite}}=\frac{\mathrm{CRP}}{\text{Neutrophils}+1}\;\text{and}\;{∆}_{\text{ratio}}=\frac{\mathrm{CRP}}{\text{Albumin}}$$ (10)

These variables approximate biological cascades and antagonisms modeled in the SDDE system. Importantly, these ratios are derived from single baseline measurements and do not represent observed temporal trajectories. They should therefore be interpreted as functional summaries of interbiomarker imbalance or misalignment rather than true physiological delays measured over time. Higher values suggest either amplification (high CRP versus neutrophils) or failure of compensatory regulation (low albumin relative to inflammation).

#### Heart failure outcome

Incident heart failure events and time-to-event (in days from baseline) were obtained from hospital inpatient records using ICD-10 diagnostic codes.

#### Covariates

Classical cardiovascular risk factors, including age, sex, previous cardiovascular events, body mass index (BMI), hypertension, diabetes, dyslipidemia, and smoking status, were included as covariates.

Participants were categorized by self-report, as “current”, “past”, or “never” smokers. Current tobacco smokers were defined as participants who responded “yes, on most or all days” or “yes, only occasionally” to the question: “Do you smoke tobacco now?”

CVD were defined by heart attack, angina, and stroke, as diagnosed by a doctor and reported in questionnaires.

BMI was calculated by dividing weight (in kilograms) by height squared (in meters).

Hypertension was defined as either by the use of antihypertensive medication, having a systolic blood pressure of at least 140 mm Hg and/or a diastolic blood pressure of at least 90 mm Hg. This definition aligns with the guidelines set by the European Society of Cardiology and includes cases managed with antihypertensive drugs or diagnosed by a doctor.

Diabetes status was determined either using antidiabetic medication, a doctor’s diagnosis of diabetes, or a fasting glucose concentration of ≥7 mmol/l.

Dyslipidemia was identified if an individual had a fasting plasma total cholesterol level of ≥6.61 mmol/l, a low-density lipoprotein cholesterol of ≥4.1 mmol/l, and a triglyceride level of >1.7 mmol/l or if they were taking statin medication. Medication usage was assessed through the question, “Do you regularly take any of the following medications?”.

#### Statistical analysis

Characteristics of the study population were described as the means with SD for continuous variables. Categorical variables were described as numbers and proportions. Comparisons between groups were performed using Student’s *t* test for continuous variables, and Pearson’s *χ*^2^ test was performed for categorical variables.

Adjusted Cox proportional hazards regression model was used to estimate the hazard ratio (HR) and 95% confidence intervals (CIs) for the association between heart failure and *τ*_composite_ and $\Delta_{\text{ratio}}$. Kaplan–Meier analysis was performed and compared by log-rank test. Follow-up time for each participant was calculated as the difference between the examination date in the UK Biobank and the last known event (2020 December 19) or censored from the linked CVD life. The median follow-up was 11.89 (interquartile range [IQR], 11.19 to 12.58) years.

As a sensitivity analysis, additional Cox models were constructed including the individual biomarker components (CRP, neutrophils, and albumin) entered separately, without ratio transformation. These models were compared to those including delay-based proxy ratios (CRP/neutrophils and CRP/albumin) to contextualize the added value of integrated delay metrics beyond single-marker effects.

Cox models were adjusted for age, gender, BMI, hypertension, diabetes, dyslipidemia, previous CVD, and tobacco smoking status. “Patient with no heart failure event” was considered as the referent group in the analyses. The proportional hazards assumption was assessed for all Cox models using Schoenfeld residuals and time-by-covariate interaction terms. No major violations of the proportional hazards assumption were detected for the delay-based proxy variables.

Statistics were performed using SAS software (version 9.4; SAS Institute, Carry, NC). A *P* < 0.05 was considered statistically significant.

#### Results of data analysis

##### Real-world observational results: UK Biobank cohort

Baseline characteristics of the study population are shown in Table 1.

**Table 1.** Descriptive characteristics of the study population

Characteristic	Reference group (Event_CV = 0)		Heart failure (Event_CV = 1)		*P* value
	*N* = 495,533		*N* = 6,945		
	Mean	SD	Mean	SD	
Age	56.45	8.09	61.79	6.35	<0.001
BMI	27.39	4.77	29.78	5.86	<0.001
CRP	2.58	4.32	4.11	6.14	<0.001
$\tau_{\text{composite}}$	0.48	0.77	0.69	0.98	<0.001
${∆}_{\text{ratio}}$	0.06	0.10	0.09	0.15	<0.001
Neutrophils	4.22	1.42	4.77	1.68	<0.001
Albumin	45.22	2.62	44.44	2.79	<0.001
	***N***	**%**	***N***	**%**	
Females	270,859	54.66%	2,507	36.10%	<0.001
Hypertension	237,445	48.01%	5,273	76.07%	<0.001
Previous CVD	27,091	5.48%	2,069	29.89%	<0.001
Diabetes	32,100	7.51%	1,344	22.06%	<0.001
Dyslipidemia	270,040	57.71%	5,101	76.91%	<0.001
Tobacco smoking					<0.001
Current	51,940	10.54%	1,034	15.04%	
Past	169,923	34.49%	3,127	45.48%	
Never	270,791	54.97%	2,714	39.48%	

Categorical variables are reported as *N* (%), and continuous variables are reported as means ± standard deviation (SD). *P* values were computed using *χ*^2^ or Fisher’s exact tests for categorical variables and Student’s *t* test or Wilcoxon rank-sum test for continuous variables, as appropriate.

**Fig. 5.**
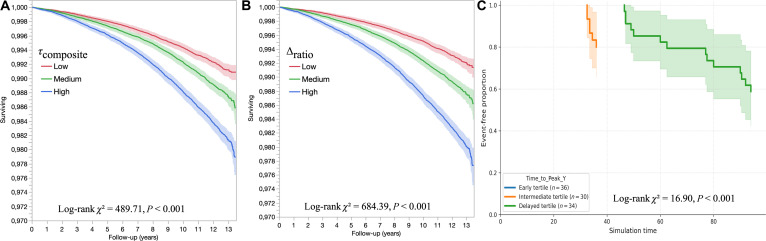
Kaplan–Meier survival curves illustrating event-free trajectories stratified by delay-based markers in real and virtual populations. (A and B) Cardiovascular-event-free survival in the UK Biobank cohort stratified by tertiles of $\tau_{\text{composite}}$ (CRP/[neutrophils + 1]) and $\Delta_{\text{ratio}}$ (CRP/albumin), respectively. These observational associations reflect delay-related inflammatory patterns and provide epidemiological consistency with the SDDE-derived hypothesis, without implying mechanistic equivalence or causal inference. (C) An analogous analysis in the SDDE-simulated virtual population, where individuals are stratified by tertiles of Time_to_Peak_Y, a model-derived measure capturing the temporal delay in the latent clinical output *Y*(*t*). Event times correspond to the simulated time at which *Y*(*t*) reaches its maximum for event-positive individuals, while nonevent trajectories are censored at the end of the simulation window. In this panel, separation between curves should be interpreted as a temporal reorganization of event emergence driven by delayed dynamics within the model rather than as a measure of predictive performance or absolute risk. Earlier tertiles correspond to trajectories with rapid stabilization and low event incidence, whereas delayed tertiles exhibit later but more frequent event occurrence. This pattern illustrates how delay structure alone can shape event timing in a controlled dynamical system, providing a conceptual analog to the observational stratifications observed in real-world data.

Participants exhibiting a higher composite delay signal, through interbiomarker misalignment (e.g., CRP versus neutrophils or albumin), had a significantly increased risk of developing incident heart failure. Over a median follow-up of 11.89 years (IQR, 11.19 to 12.58), Kaplan–Meier survival curves stratified by $\tau_{\text{composite}}$ and $\Delta_{\text{ratio}}$ tertiles showed early and sustained divergence (for $\tau_{\text{composite}}$: log-rank *χ*^2^ = 489.71, *P* < 0.001; Fig. 5A; and for $\Delta_{\text{ratio}}$: log-rank *χ*^2^ = 684.39, *P* < 0.001; Fig. 5B).

In exploratory models, additional delay-related biomarkers were tested. Specifically, the CRP-to-neutrophil ratio ($\tau_{\text{composite}}$) and the CRP-to-albumin ratio ($\Delta_{\text{ratio}}$) were both significantly associated with increased risk of heart failure (*P* < 0.01), reinforcing the SDDE-derived hypothesis that biological delays and regulatory imbalance can be clinically relevant markers of risk. The full multivariable model is detailed in Table 2.

**Table 2.** Adjusted Cox regression model for heart failure events

Parameters	HR	95% CI	*P* value
Age	1.09	1.08–1.10	<0.001
BMI	1.06	1.04–1.07	<0.001
$\tau_{\text{composite}}$	1.06	1.05–1.07	<0.001
Δ_ratio_	1.02	1.01–1.03	0.002
Females	0.68	0.64–0.73	<0.001
Hypertension	1.61	1.37–1.74	<0.001
Previous CVD	2.54	2.36–2.72	<0.001
Diabetes	1.46	1.35–1.58	<0.001
Dyslipidemia	1.02	0.95–1.09	0.561
Tobacco			<0.001
Current	1.88	1.72–2.05	<0.001
Past	1.21	1.13–1.29	<0.001
Never	Ref.	–	–

For categorical variables, hazard ratios (HRs) are reported relative to a reference category (Ref.), which is indicated in the table (i.e., tobacco).

When included alongside their component variables (e.g., CRP, neutrophils, and albumin), the delay proxies ($\tau_{\text{composite}}$ and $\Delta_{\text{ratio}}$) remained statistically significant but exhibited attenuated effect sizes and Wald statistics. In sensitivity analyses including CRP, neutrophils, and albumin simultaneously as separate covariates, the delay-based ratios remained associated with incident heart failure, although effect sizes were attenuated because of partial collinearity. This finding suggests that while individual inflammatory markers contribute to risk, integrated delay proxies may capture higher-order physiological relationships not fully represented by any single biomarker alone.

This suggests partial redundancy and collinearity, which may dilute interpretability. These findings imply that integrated delay metrics may capture relevant physiological dynamics more robustly than any single inflammatory marker alone, especially when modeling complex multiscale interactions.

These findings suggest that interbiomarker misalignment and regulatory imbalance, captured through delay-like proxy ratios, can serve as clinically meaningful predictors of long-term cardiovascular vulnerability. This real-world evidence reinforces the simulation-derived conclusion that delayed, multiscale physiological interactions shape risk emergence in stochastic systems.

## Discussion

This study integrates mechanistic modeling and epidemiological validation to demonstrate that time-lagged inflammatory dynamics, as captured by SDDEs, are potent predictors of cardiovascular outcomes. By incorporating both temporal delays and stochastic fluctuations, the digital twin framework enhances the physiological realism of disease modeling and opens new avenues for personalized risk prediction [18,27]. Importantly, sensitivity analyses incorporating individual biomarker components confirmed that the observed associations were not solely driven by CRP, neutrophils, or albumin considered in isolation but rather by their relative imbalance, consistent with the SDDE-derived hypothesis of delayed and dysregulated inflammatory dynamics. The delay proxies used in the epidemiological analysis (CRP-to-neutrophil and CRP-to-albumin ratios) do not represent measured temporal delays. Instead, they should be understood as structural analogs of delayed dynamics, summarizing the relative positioning of upstream inflammatory activation and downstream regulatory responses at a single time point. Elevated CRP-to-neutrophil ratios may reflect sustained or dysregulated inflammatory burden, while CRP-to-albumin ratios may capture a combination of inflammation, hepatic function, nutritional status, and catabolic state. These mechanisms are not mutually exclusive with delayed dynamics and may represent different facets of the same underlying pathophysiological process.

Throughout this work, simulation-based and observational results are deliberately presented as complementary but conceptually distinct lines of evidence: The former explore mechanistic plausibility within a mathematical system, while the latter document associations in real-world data.

Delayed responses are a fundamental feature of biological systems. Inflammatory processes unfold across multiple timescales: Neutrophils respond rapidly, while downstream markers like CRP rise more gradually, and albumin reflects slower regulatory feedback [8,9,28]. This temporal sequencing is not only biologically plausible but also appears to encode risk. The simulations show that delays, especially when paired with antagonistic regulatory dynamics, can differentiate trajectories within the model once the outcome is defined endogenously, illustrating how delay may function as a risk-defining feature in delayed stochastic systems. These results align with systems biology models, where delayed feedback loops are known to underlie critical transitions and tipping points in complex systems [15,16].

It is essential to distinguish model-internal causality, which governs the behavior of the SDDE system, from biological causality, which cannot be inferred from simulations or observational data alone. Accordingly, the SDDE simulations should be interpreted as hypothesis-generating instruments that explore how delayed feedback and stochasticity can organize risk within a mathematical system rather than as mechanistic proof of causal pathways in human disease.

The SDDE framework led to near-complete stratification in logistic regression under idealized simulation conditions. This finding suggests that temporal delay functions as a structurally sufficient feature within the model, supporting a system-level plausibility hypothesis rather than establishing biological causality, a concept supported by mathematical modeling of immune and neuroendocrine feedback [15,29]. In this sense, the SDDE-based digital twin used here should be understood as a conceptual modeling construct rather than an operational clinical digital twin.

Furthermore, the incorporation of stochastic noise captures the intrinsic variability of biological systems [20,21,30]. Variability in immune response, gene expression, and metabolic function is well documented and arises from both molecular and environmental fluctuations [10,13,19]. SDDEs offer a flexible framework to simulate these phenomena, and in this model, identical stimuli led to divergent clinical outcomes, reflecting the heterogeneity observed in real-world patient populations [31]. Incorporating parameter heterogeneity would further align the SDDE framework with known immunological diversity, enabling exploration of how genetic background, sex, and immune aging modulate delayed inflammatory responses.

Associations observed in the UK Biobank supported the epidemiological consistency of this framework. Delay-based biomarkers such as CRP-to-neutrophil and CRP-to-albumin ratios, conceptual proxies of delayed, and dysregulated inflammation were robustly associated with incident heart failure over nearly 12 years of follow-up. These associations persisted after adjustment for traditional risk factors. While CRP and albumin have individually been associated with cardiovascular outcomes [17,32], the novelty of this approach lies in demonstrating that temporal misalignment between these markers encodes additional mechanistic information.

From a clinical perspective, these findings support the hypothesis that inflammatory timing, not just inflammatory burden, shapes cardiovascular vulnerability. This is particularly relevant in the context of inflamm-aging, a chronic, low-grade, and often delayed inflammatory state linked to aging and chronic disease risk [28].

### Future directions

To maximize the usability and broader impact of the proposed framework, we plan to develop a user-friendly interactive exploration tool based on this work. This resource will enable users to visualize and interrogate how variations in delay, stochasticity, and regulatory balance influence latent cardio-inflammatory trajectories within the SDDE framework. Consistent with the conceptual digital twin positioning adopted here, the tool will be designed for hypothesis exploration, education, and model interpretation rather than for clinical decision support or real-time deployment.

### Limitations

Several limitations should be acknowledged in this study. The delay proxies used in the epidemiological analysis are indirect approximations of true temporal dynamics, reflecting cross-sectional imbalance rather than observed time-lagged biomarker kinetics. These ratios reflect relative biomarker levels at a single time point rather than dynamically observed lags. While informative, they cannot fully capture the individual time courses of inflammatory activation and resolution. Longitudinal biomarker data, especially with repeated high-frequency sampling, would allow more precise estimation of physiological delays and validation of the simulated SDDE outputs. Future studies combining repeated biomarker measurements or high-frequency inflammatory profiling could directly test whether these proxy ratios correlate with empirically observed delayed inflammatory kinetics.

The simulation model uses fixed structural and kinetic parameters across all virtual individuals, with interindividual variability arising solely from stochastic noise. While this design allows isolation of the effects of delay and stochasticity, it underrepresents key dimensions of biological heterogeneity, including genetic diversity, sex-specific immune responses, baseline inflammatory phenotypes, and immune senescence. From an immunoinformatics perspective, such sources of variability are known to substantially shape inflammatory dynamics and cardiovascular vulnerability. In reality, biological responses vary markedly between individuals due to genetics, hormonal states, comorbidities, and environmental exposures [19]. Although stochasticity introduces some interindividual variability, future models should incorporate hierarchical or Bayesian frameworks to reflect parameter heterogeneity and to personalize predictions.

Future extensions of this framework could explicitly incorporate parameter heterogeneity through hierarchical or Bayesian SDDE formulations, allowing subject-specific delay distributions and gain parameters to be inferred or sampled. Such approaches would enable the integration of sex-stratified dynamics, immune-age-dependent delays, and baseline inflammatory states, thereby enhancing both immunological realism and personalization of digital twin simulations.

The noise processes modeled in SDDEs assume Gaussian (white) noise, which is mathematically tractable but may not represent the full complexity of biological randomness. Empirical studies show that noise in gene regulation, immune signaling, and metabolic control may follow burst-like, non-Gaussian, or temporally correlated patterns [33,34]. Integrating colored noise or jump processes could enhance physiological realism in future models.

Then, although this model focuses on inflammation, other physiological systems (e.g., autonomic regulation, neuroendocrine rhythms, and microbiome dynamics) may introduce additional delays and feedback loops that influence cardiovascular outcomes [7]. Integrating such multiscale systems into future digital twin models may yield more complete representations of disease emergence.

## Conclusion

This study demonstrates that delayed inflammatory dynamics can structure risk trajectories within delayed stochastic systems, emerging from the sequential interactions between neutrophils, CRP, and albumin, can shape risk trajectories within delayed stochastic systems and motivate delay-aware hypothesis generation in clinical modeling under stochastic conditions. Through SDDE simulation, this work shows that time-to-peak of the latent clinical output *Y*(*t*) is a mechanistic and quantifiable marker of vulnerability within the SDDE framework, capable of structuring risk gradients within delayed stochastic systems under idealized conditions. These simulation-based insights were supported by consistent associations in a large-scale population cohort, where simple delay proxies derived from baseline biomarkers (e.g., CRP/neutrophils and CRP/albumin) were significantly associated with incident heart failure over long-term follow-up.

Together, these findings suggest that time-aware features, especially those reflecting regulatory delays, should be considered as physiologically meaningful predictors in personalized risk modeling. The SDDE framework offers a powerful mathematical approach to embedding such dynamics into digital twin architectures, enhancing their capacity to simulate real-world disease trajectories and optimize individualized prevention. This approach lays the foundation for delay-aware digital twins, capable of modeling individual disease trajectories with temporal fidelity. By embedding physiologically realistic delay and feedback into personalized simulations, SDDE-based digital twins move beyond static predictors toward more dynamic, actionable models of clinical risk.

## Data Availability

UK Biobank data are available through the UK Biobank Access Management System (http://www.ukbiobank.ac.uk/register-apply/). For data access, responsibility, and analysis, A.V. had full access to all the data in the study and takes responsibility for the integrity of the data and the accuracy of the data analysis.
