# Supplementary material for: Predicting Cardiovascular Events with Time-Lagged Inflammatory Dynamics: Stochastic Delay Modeling
Source: Comput Struct Biotechnol J. 2026 Mar 18;35(1):0005. doi: 10.34133/csbj.0005 (PMC13068005; doi:10.34133/csbj.0005)

**Supplementary Table S2: Sensitivity of delay-based discrimination to event threshold definition.**

| Event prevalence (%) | Threshold definition | Max_Y threshold | AUC (Time_to_Peak_Y → Event_CV) | Separation |
| --- | --- | --- | --- | --- |
| 15 | Max_Y > Q85 | 130.87 | 0.78 | Partial |
| 20 | Max_Y > Q80 | 116.851 | 0.797 | Partial |
| 30 | Max_Y > Q70 | 104.039 | 0.811 | Partial |

Event definitions were varied to generate simulated cardiovascular event prevalences of 15%, 20%, and 30%, corresponding to different quantiles of the Max_Y distribution. Discriminative performance of Time_to_Peak_Y was assessed using the area under the ROC curve (AUC). While discrimination remained robust across thresholds, perfect separation was not systematically observed, indicating that sharp separation depends on the specific event definition rather than reflecting intrinsic biological determinism.

**Supplementary Figure S1: Effect of independent outcome noise on delay-based discrimination.**

Independent Gaussian noise was added to the outcome variable at the event-definition stage before thresholding. This perturbation removed perfect separation between delayed and early trajectories, while preserving a strong association between Time_to_Peak_Y and simulated cardiovascular events. These results demonstrate that the delay-based signal is not solely driven by deterministic outcome construction.


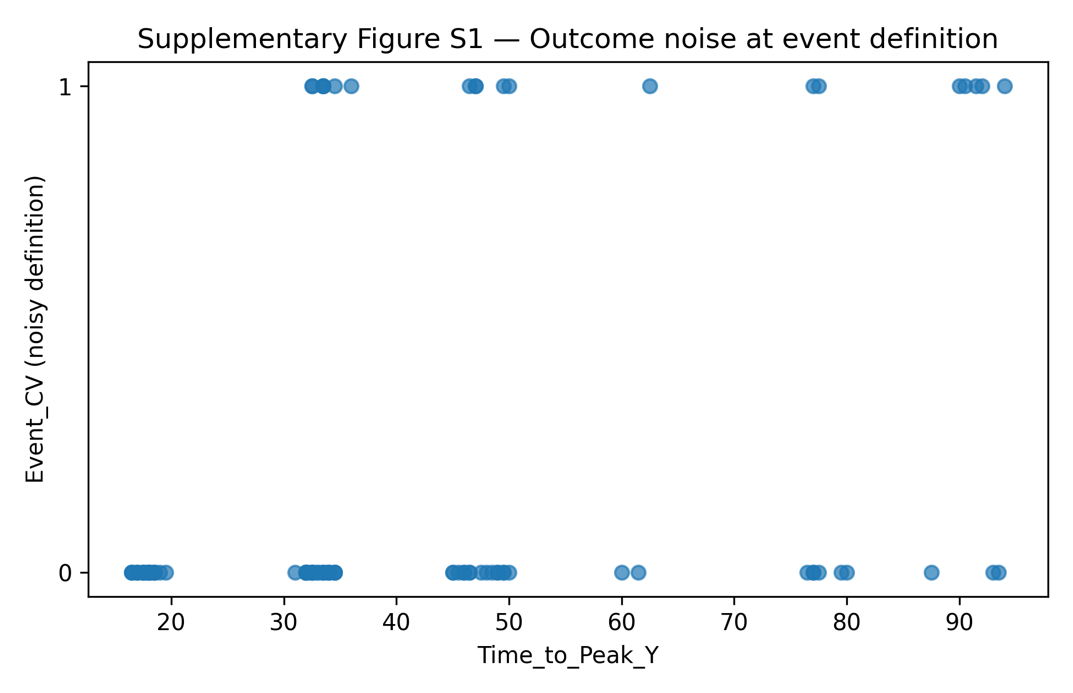


**Supplementary Figure S2: Impact of inter-individual parameter heterogeneity on delay-based separation.**

Moderate inter-individual variability (10% coefficient of variation) was introduced as a proxy for parameter heterogeneity, affecting temporal response characteristics. Parameter heterogeneity reduced the sharpness of separation between event and non-event groups but preserved the global relationship between delayed responses and simulated cardiovascular events, supporting the structural robustness of delay-based effects.


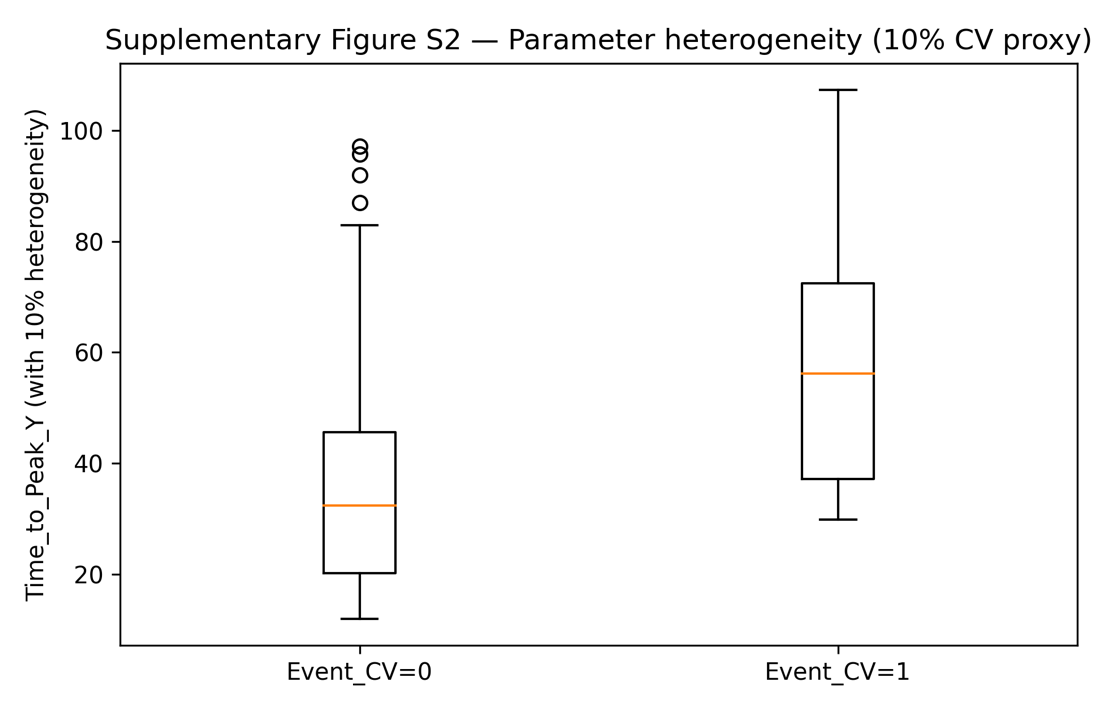

Supplement: Supplementary 1 — Figs. S1 and S2 Tables S1 and S2 [file csbj.0005.f1.zip › supplementary_tables_and_figures.docx]
